# Species Identification and Genetic Diversity Analysis of Medicinal Plants *Aconitum pendulum* Busch and *Aconitum flavum* Hand.-Mazz.

**DOI:** 10.3390/plants13060885

**Published:** 2024-03-19

**Authors:** Jing Sun, Qing Sun, Xin Li, Wenjing Li, Yi Li, Yubi Zhou, Yanping Hu

**Affiliations:** 1 Qinghai Provincial Key Laboratory of Qinghai–Tibet Plateau Biological Resources, Northwest Institute of Plateau Biology, Chinese Academy of Sciences, Xining 810008, China; sunjing21@mails.ucas.ac.cn (J.S.); sunqing22@mails.ucas.ac.cn (Q.S.); lixin2311@mails.ucas.ac.cn (X.L.); liyi@nwipb.cas.cn (Y.L.); ybzhou@nwipb.cas.cn (Y.Z.); 2 University of Chinese Academy of Sciences, Beijing 100049, China; 3 Scientific Research and Popularization Base of Qinghai–Tibet Plateau Biology, Qinghai Provincial Key Laboratory of Animal Ecological Genomics, Xining 810008, China; wjli@nwipb.cas.cn

**Keywords:** *Aconitum pendulum*, *Aconitum flavum*, pubescence on the inflorescence, inter-simple sequence repeat (ISSR), cluster analysis, species identification

## Abstract

The classification system for the genus *Aconitum* is highly complex. It is also the subject of ongoing debate. *Aconitum pendulum* Busch and *Aconitum flavum* Hand.-Mazz. are perennial herbs of the genus *Aconitum*. Dried roots of these two plants are used in traditional Chinese medicine. In this study, morphological observations and ISSR molecular markers were employed to discriminate between *A. flavum* and *A. pendulum*, with the objective of gaining insights into the interspecies classification of *Aconitum*. The pubescence on the inflorescence of *A. flavum* was found to be appressed, while that on the inflorescence of *A. pendulum* was spread. UPGMA (unweighted pair-group method with arithmetic average) cluster analysis, PCoA (principal coordinates analysis), and Bayesian structural analysis divided the 199 individuals (99 individuals from DWM population and 100 individuals from QHL population) into two main branches, which is consistent with the observations of the morphology of pubescence on the inflorescence. These analyses indicated that *A. flavum* and *A. pendulum* are distinct species. No diagnostic bands were found between the two species. Two primer combinations (UBC808 and UBC853) were ultimately selected for species identification of *A. flavum* and *A. pendulum*. This study revealed high levels of genetic diversity in both *A. flavum* (*H*_e_ = 0.254, *I* = 0.395, *PPB* = 95.85%) and *A. pendulum* (*H*_e_ = 0.291, *I* = 0.445, *PPB* = 94.58%). We may say, therefore, that ISSR molecular markers are useful for distinguishing *A. flavum* and *A. pendulum*, and they are also suitable for revealing genetic diversity and population structure.

## 1. Introduction

The genus *Aconitum*, which belongs to the Ranunculaceae family, occurs in temperate regions of the Northern Hemisphere. This genus encompasses about 400 species, including 211 species that are distributed in China, of which 166 are endemic [1]. Despite the fact that most *Aconitum* species are poisonous, many species have been utilized for medicinal purposes [2,3]. *Aconitum pendulum* Busch and *Aconitum flavum* Hand.-Mazz. are perennial herbaceous plants of the genus *Aconitum*. They are mainly distributed in Qinghai, Gansu, Nei Mongol, southern Ningxia, northwestern Sichuan, and northern Tibet. They grow on grassy slopes and in forest margins at altitudes of 2000–3700 m [1]. *A. flavum* and *A. pendulum* are the dominant toxic weeds in the alpine meadows of the Qinghai–Tibet Plateau [4]. They are known to exhibit allelopathic effects. Secretions from their roots, stems, and leaves inhibit the growth of other plants and thus pose a certain level of threat to grassland ecology and to the development of animal husbandry [5]. Additionally, the dried roots of these two plants are used to make one of the most famous Tibetan medicines, namely, Tiebangchui (TBC). To date, a total of 84 alkaloids have been obtained from TBC [6]. Diterpenoid alkaloids, such as aconitine and 3-acetylaconitine, are the characteristic compounds in most of the *Aconitum* species [7]. These phytochemicals have been shown to exhibit a wide range of pharmacological properties, such as dispelling cold, alleviating pain, and inhibiting bacteria, as well as treating injuries, rheumatic lumbar pain, and chilblains [8,9].

More than 20 *Aconitum* species are used as sources of TBC because of their morphological similarities and species diversity [6]. To avoid species confusion, only *A. flavum* and *A. pendulum* have been approved by the Chinese Pharmacopoeia Commission; these are now listed in the Pharmacopoeia Standards of Ministry of Health of the People’s Republic of China: Tibetan medicine and Qiang and Hui medicines [10,11]. At present, the artificial domestication of *Aconitum* species is still in its early stages, and commercial demand for *A. flavum* and *A. pendulum* is being met mainly by wild resources. With the development and production of new drugs, demand for these species is increasing year by year, and wild resources are declining dramatically due to excessive collection [12]. In addition, because of the need to ensure stability in clinical efficacy, the adulteration of *A. flavum* and *A. pendulum* is also a matter of increasing importance today. The hairs on the pedicels are often considered to be among the most important taxonomic characteristics within the *Aconitum* genus. In the classification of *Aconitum* species, much attention has been paid to the different types of hairs on the pedicels [13]. These hairs have been considered important evidence by researchers speculating on the origin of hybrids [14]. *A. flavum* and *A. pendulum* are closely related in terms of their morphologies [15], and species delimitation is difficult to achieve using the naked eye. Traditionally, morphological characteristics have been utilized for species classification within the *Aconitum* genus. However, more accurate identifications and phylogenetic analyses of closely related species can be obtained using molecular methods [16].

Studies on *A. flavum* and *A. pendulum* have mainly focused on their chemical composition, pharmacological properties, and toxic effects [12,17]. Consequently, little is known regarding their species differentiation and genetic diversity. Inter-simple sequence repeat (ISSR) has been widely used for genetic diversity and plant breeding in various plant species [18,19,20,21], including *Mentha* L. [22], *Hypericum* L. [23], *Ammopiptanthus nanus* [24], and *Habenaria dentata* [25]. ISSR utilizes primers with designs based on the repeating motif (microsatellite) of the eukaryotic genome, without prior knowledge of the DNA sequence [26,27]. Because of the higher annealing temperatures and longer sequences of ISSR primers, they exhibit good stability and high polymorphism. ISSR might also be used to address taxonomic and systematic issues [22,28]. Cheng et al. [29] demonstrated that ISSR was more effective in identifying *Alcea* species. ISSR was also used by Akhta et al. [30] to identify different species of the genus *Jasminum* from Pakistan. In addition, ISSR has been employed to investigate genetic diversity in *Aconitum* species such as *A. kongboense* L. [31], *A. leucostomum* Worosch [32], and *A. carmichaeli* [33]. Although the two *Aconitum* species have medicinal and commercial value, they are often used without species identification. The ability to distinguish the two species could result in improved medicinal potential, quality control, and stability in commercial products containing material from these plants. However, to the best of our knowledge, there have been no published reports on the genetic diversity in *A. flavum* and *A. pendulum*.

Due to the apparently similar morphologies and the overlapping geographical distributions of *A. flavum* and *A. pendulum*, the boundary between the two species is unclear. Increasingly, researchers are asking whether the two species might be the same. Based on the ITS sequence, Luo et al. [34] showed that *A. flavum* and *A. pendulum* were not sister groups. In contrast, *A. flavum* and *A. pendulum* were identified as sister groups from 25 morphological characters by the authors of Cui et al. [35]. After conducting chloroplast genome and whole-genome SNP analyses, Li [36] proposed that *A. flavum* and *A. pendulum* should be merged into one species. Additionally, Ren [37] recommended that *A. hezuoense* and *A. lianhuashanicum* should be treated as synonyms for *A. flavum*. In light of the previous research, we sought to prove whether or not *A. flavum* and *A. pendulum* are the same species by means of morphological observation and molecular analysis.

The main objectives of this study were as follows: (1) to identify *A. flavum* and *A. pendulum* by observing morphologies of pubescence on inflorescence characters and ISSR markers, and (2) to assess genetic diversity and genetic variation in *A. flavum* and *A. pendulum*.

## 2. Results

### 2.1. Identification of Morphologies of Pubescence on Inflorescence

**Figure 1:**
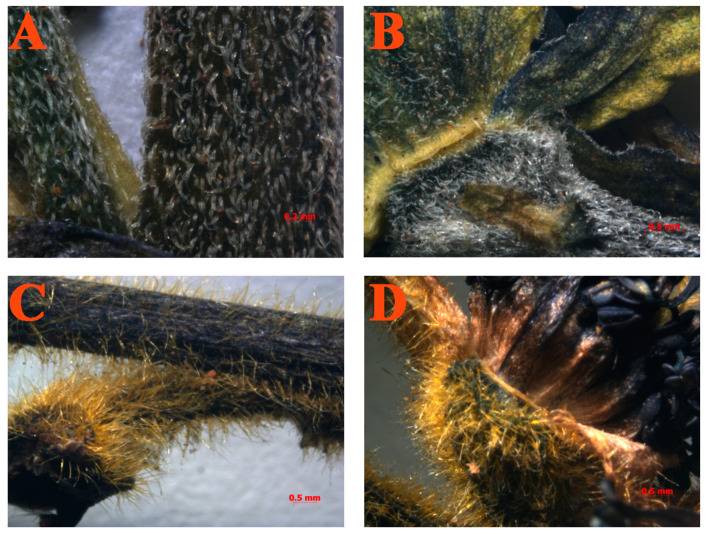
Hairs on the rachis and pedicels of *A. flavum* and *A. pendulum*: (**A**) rachis of *A. flavum*; (**B**) pedicels of *A. flavum*; (**C**) rachis of *A. pendulum*; (**D**) pedicels of *A. pendulum*.

In the classification of the genus *Aconitum*, hairs on the pedicels are often regarded as important taxonomic traits. In the present study, to identify *A. flavum* and *A. pendulum*, morphologies of the hairs on the rachis and pedicels were observed with a stereoscopic microscope (Figure 1). We found that the rachis and pedicels of *A. flavum* are covered with densely appressed pubescence, which are generally white in color. The stem of *A. flavum* is basally glabrous; it is also apically retrorse and appressed pubescent. In *A. pendulum*, the rachis and pedicels have densely spread pubescence, which are yellow in color. The stem of *A. pendulum* is basally glabrous; it is also apically sparse and spreading yellow pubescent. The most important difference between these two species concerns the spreading hairs in inflorescence. In *A. flavum*, these are appressed; in *A. pendulum*, they are spread. During specimen observation, no intermediate forms were found.

### 2.2. Individual Identification and Validation

**Figure 2:**
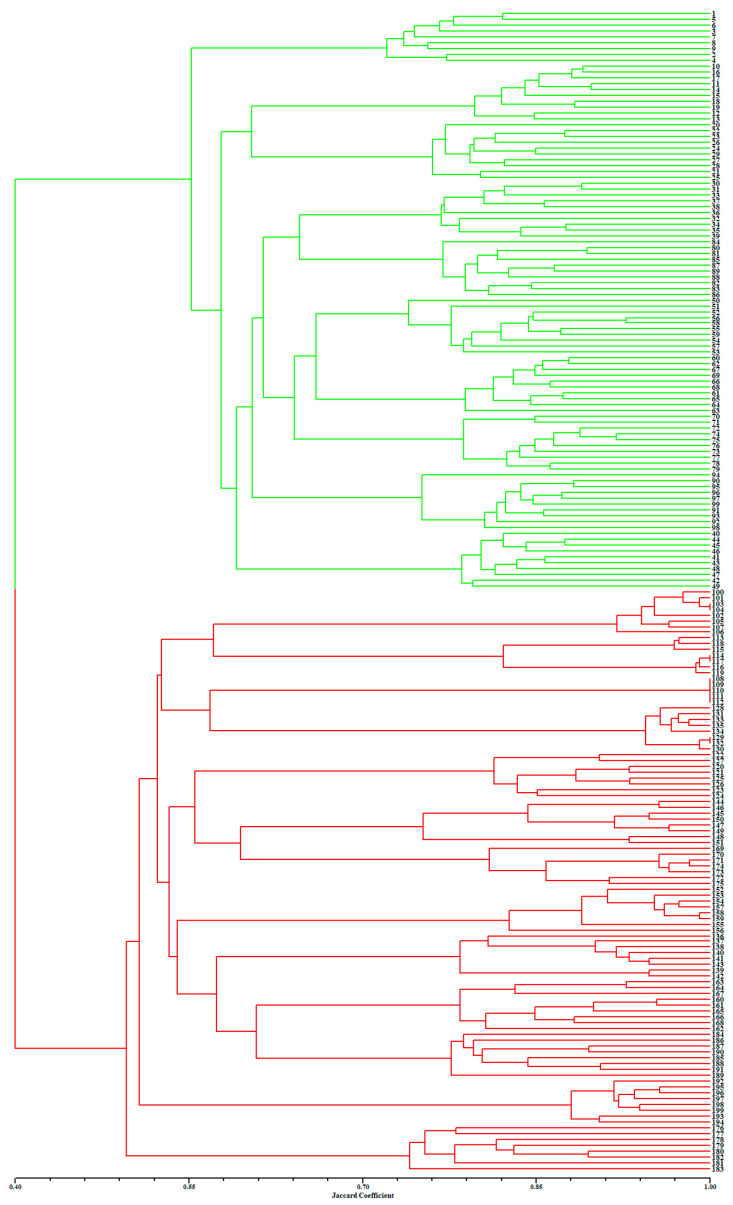
Results of UPGMA clustering based on 11 ISSR primers, performed on 199 individuals of *A. flavum* and *A. pendulum*: green represents *A. flavum* individuals (1–99); red represents *A. pendulum* individuals (100–199).

**Figure 3:**
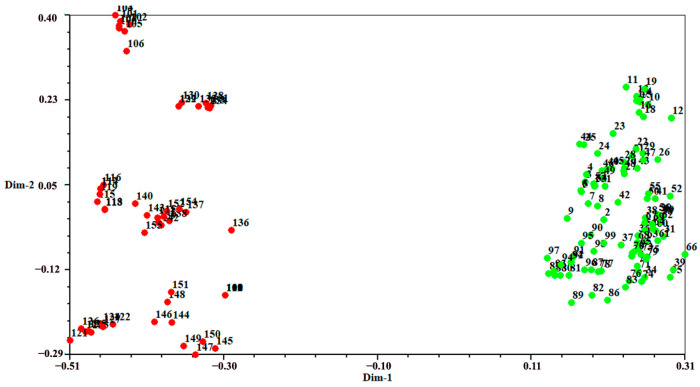
Principal coordinates plot showing patterns of separation among 199 individuals of *A. flavum* and *A. pendulum*: green represents *A. flavum* individuals (1–99); red represents *A. pendulum* individuals (100–199).

**Figure 4:**
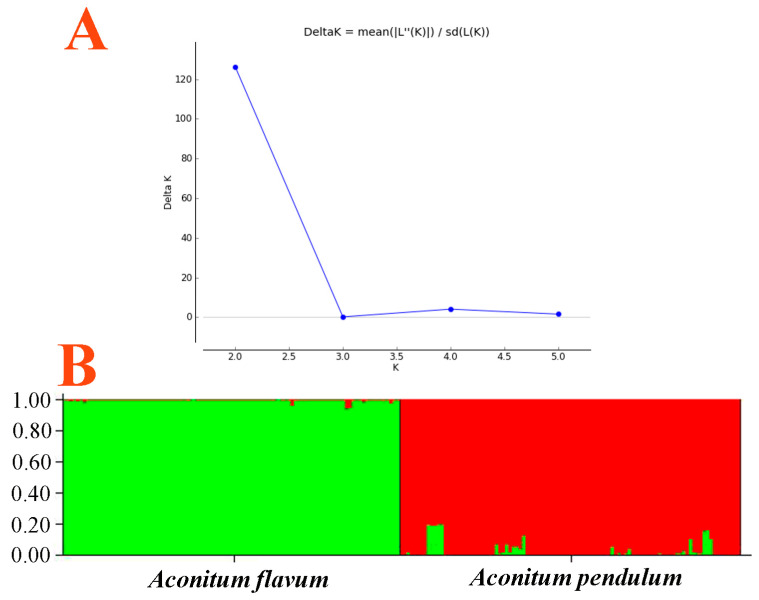
Bayesian inference of the number of clusters (*K*) in *A. flavum* and *A. pendulum*: (**A**) *K* was estimated from plots of ad hoc posterior probability models of *ΔK*; (**B**) Bayesian admixture proportions (*q*) of individuals of *A. flavum* and *A. pendulum* for *K* = 2.

Figure 2 presents a dendrogram obtained using the unweighted pair-group method with arithmetic average (UPGMA), based on a Jaccard genetic similarity coefficient and 11 ISSR primers. The individuals were divided into two groups. Group I included 99 individuals of *A. flavum*; group II contained all 100 individuals of *A. pendulum*. The genetic relationships among 199 genotypes were also visualized using PCoA (principal coordinates analysis); the extent of the relatedness and disparity among the genotypes is shown in Figure 3. The PCoA plot produced two major clusters which were clearly separated, and this plot supported the results of the UPGMA cluster analysis. In addition, the relationships among individuals were further examined using STRUCTURE (Figure 4), and similar results were obtained. Bayesian cluster analysis partitioned all the individuals into two distinct groups (Figure 4B). The highest peak in *ΔK* revealed the best value for *K* = 2 (*ΔK* = 126.151) (Figure 4A). This was confirmed by results obtained using BAPS (Bayesian analysis of population structure). The number of clusters in optimal partition assignment with BAPS was determined as *K* = 2 (Figure S1), with a log marginal likelihood of optimal partition of −32,586.38, and with posterior probability reaching its highest value (~1). The results of the Bayesian cluster analysis were in line with the UPGMA clustering and the PCoA plot. Taken together, the results showed that the two species (*A. flavum* and *A. pendulum*) could be clearly divided into two branches via ISSR molecular markers. We may say, therefore, that ISSR molecular primers can be used to identify individuals of these two species.

**Figure 5:**
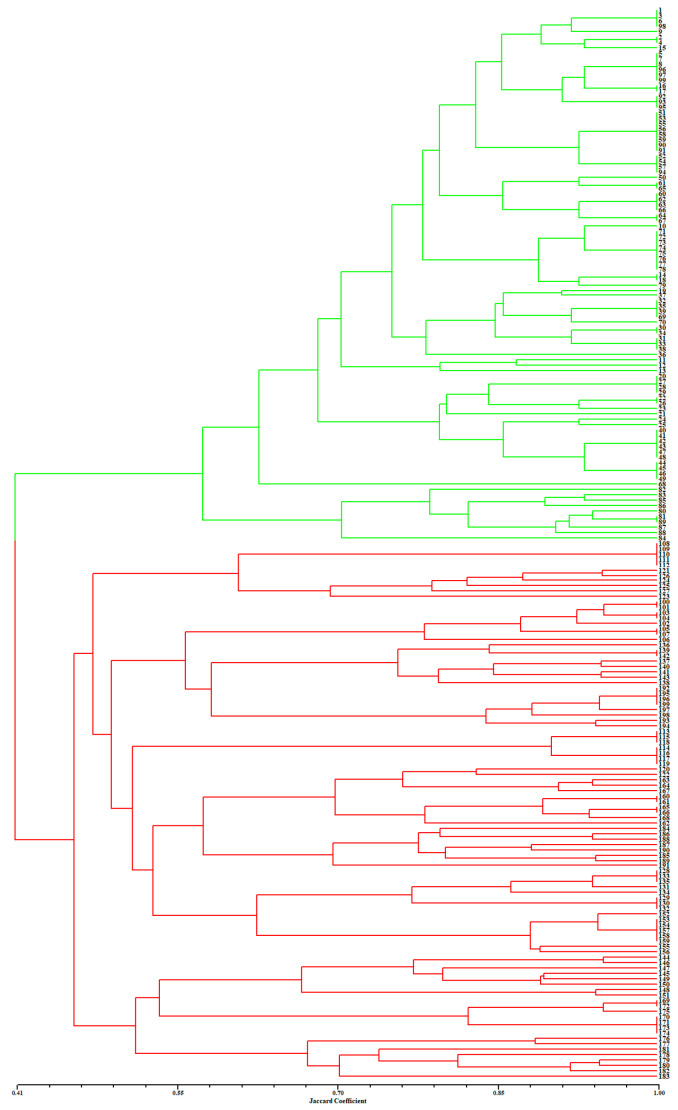
Results of UPGMA clustering of 199 individuals based on UBC808 and UBC853: green represents *A. flavum* individuals (1–99); red represents *A. pendulum* individuals (100–199).

Initially, we sought to identify distinctive bands for *A. flavum* and *A. pendulum* via the process of band selection. However, no diagnostic bands were found between these two species. More seriously still, no single specific primer was found between *A. flavum* and *A. pendulum*. Finally, the two primer combinations UBC808 and UBC853 were used to identify the two species. The UPGMA dendrogram based on the genetic similarity coefficient then revealed that all individuals could be clustered into two well-defined and separate groups (Figure 5).

Subsequently, a standard database was established using two primer combinations (UBC808 and UBC853) for species identification of *A. flavum* and *A. pendulum*. Sixty-nine blind individuals from four populations (B, BM, GQ, SL populations) were tested to determine whether they were *A. flavum* or *A. pendulum* using the established identification system.

The results indicated that the blind individuals from the B and BM populations were clustered into the branch of *A. flavum* (Figure S2A). The blind individuals from the GQ and SL populations were clustered into the branch of *A. pendulum* (Figure S2B). This further confirmed that the selected primer combinations (UBC808 and UBC853) could effectively identify *A. flavum* and *A. pendulum* using ISSR molecular markers.

### 2.3. ISSR Genetic Diversity Analysis

A total of 11 primers with high repeatability and good polymorphism were selected for ISSR–PCR amplification. Details of these primers are presented in Table S1. A photograph of gel electrophoresis was presented in Figure S3. For 135 individuals of *A. flavum*, 193 bands were amplified, with 185 polymorphic bands (*PPB* = 95.85%). The *PPB* ranged from 78.57% (UBC811) to 100% (UBC824, UBC825, UBC826, UBC846, UBC887, and UBC890). For 133 individuals of *A. pendulum*, 203 bands were amplified, of which 94.58% were polymorphic bands, resulting in an average of 17.45 polymorphic bands per primer. The *PPB* of *A. pendulum* ranged from 82.35% (UBC807) to 100% (UBC824, UBC825, UBC826, UBC846, UBC853, and UBC887).

Details of genetic diversity parameters are presented in Table 1. Genetic diversity in *A. pendulum* (*PPB* = 94.58%, *H*_e_ = 0.291 ± 0.160, *I* = 0.445 ± 0.209) was slightly higher than that in *A. flavum* (*PPB* = 95.58%, *H*_e_ = 0.254 ± 0.170, *I* = 0.395 ± 0.223). Genetic variation among *A. pendulum* populations (*G*_st_ = 0.342) was higher than among *A. flavum* populations (*G*_st_ = 0.252). Gene flow in *A. flavum* (*N*_m_ = 1.488) was higher than in *A. pendulum* (*N*_m_ = 0.961). The fixation index in *A. pendulum* (*F*_st_ = 0.375) was higher than in *A. flavum* (*F*_st_ = 0.303).

AMOVA analysis indicated that most of the genetic differentiation was distributed within populations (69.66% in the case of *A. flavum*, 62.52% in the case of *A. pendulum*) (Table S2). AMOVA testing also revealed significant differentiation among and within populations (*p* < 0.001, Table S2). A similar result was obtained from the HICKORY calculation: interspecies differentiation (*θ*_B_) was found to be 0.265 under the *f* = full model, which had the smallest DIC value (Table S3), and a corresponding *f* value of 0.554. Additionally, gene flow (*N*_m_ = 4.619) was calculated based on *G*_st_.

## 3. Discussion

The taxa of the genus *Aconitum* are considered to be complicated and controversial because of the morphological similarities among species. The relationship between *A. flavum* and *A. pendulum* is especially noteworthy in this regard. Previous studies have revealed that the main phenotypic differences between *A. flavum* and *A. pendulum* involve the spreading hairs on the rachis and pedicels [14]. In the present study, *A. flavum* and *A. pendulum* were clearly distinguished through stereomicroscopy. The spreading hairs on the inflorescence of *A. flavum* were found to be appressed, while those of *A. pendulum* were spread. This result demonstrated that these qualitative characteristics could be used to effectively differentiate between the two species. In recent years, numerous studies on the classification and phylogeny of *Aconitum* have been conducted based on ITS, cpDNA, complete plastomes, and other molecular markers [38,39,40]. *Aconitum* is generally divided into three subgenera: *A.* subgenus *Aconitum*, *A.* subgenus *Lycoctonus*, and *A.* subgenus *Gymnaconitum* [41]. Among these subgenera, *A.* subgenus *Aconitum* is the most widespread and diverse; it encompasses numerous species and exhibits complex morphological variations. Further subdivision of this subgenus therefore involves significant challenges [42]. In the *A.* subgenus *Aconitum*, *A. flavum*, *A. pendulum*, *A. liangshanicum*, and *A. brachypodum* are classified under ser. *Brachypoda* W. T. Wang. A chemical composition analysis has shown that *A. flavum*, *A. pendulum*, and *A. polyschistum* of the ser. *Brachypoda* exhibit highly evolved aconitine-type diterpenoid alkaloids, indicating a close genetic relationship between them [2]. In addition, seed morphology has revealed that the seeds of ser. *Brachypoda* have three longitudinal ridges and a relatively smooth surface, suggesting that *A. flavum* and *A. pendulum* might belong to the same natural group [43]. Furthermore, studies of the chloroplast genomes of *A. flavum* and *A. pendulum* reveal only slight differences between the two, suggesting a close genetic relationship between the two species [44,45]. All these studies demonstrated a close relationship between the two species. To date, however, no method of carrying out species identification has been reported with respect to *A. flavum* and *A. pendulum*.

Based on the ITS sequence, Luo et al. showed that *A. flavum* and *A. pendulum* are not sister groups [34]. The limited amount of DNA in the ITS may not be sufficient to resolve the phylogeny of aconites. On the other hand, ISSR molecular marker technology, which is based on the highly repetitive sequences found in eukaryotic genomes, provides additional genomic information. Due to its longer primer sequences and higher annealing temperature, ISSR can generate reliable, highly polymorphic, and reproducible amplification bands. ISSRs segregate mostly as dominant markers following simple Mendelian inheritance. However, they have also been shown to segregate as co-dominant markers in some cases, thus enabling the distinction between homozygotes and heterozygotes [21,28,30]. There are some indications that ISSR could be used to identify species. The ISSR results obtained by the authors of Boydak et al. [46] supported the idea that the well-known date palms *Phoenix theophrasti* and *P. dactylifera* are different species. The ISSR marker system has also been used to determine molecular differences between two species of cotton [28]. In light of these findings, ISSR was used in the present work to study the relationships of different individuals from *A. flavum* and *A. pendulum*. UPGMA cluster analysis, PCoA analysis, and Bayesian structural analysis divided the 199 individuals into two main branches consistent with the morphological results. There were significant genetic variations between the two species (*F*_st_ = 0.259, *p* < 0.001; Table S2). Additionally, a standard database was established by two primer combinations (UBC808 and UBC853) for species identification of *A. flavum* and *A. pendulum*. The authentications of blind individuals were identified, based on the database. The ISSR identification system for *A. flavum* and *A. pendulum* demonstrates strong accuracy.

The Qinghai–Tibet Plateau is regarded as a global biodiversity hotspot on account of its abundant biological diversity. Genetic diversity is fundamental for biodiversity, and protecting genetic diversity is crucial for the long-term survival of any species in a constantly changing environment [47,48]. ISSR molecular markers can be used to estimate genetic diversity and genetic structure within and between species. In the present study, 135 samples of *A. flavum* and 133 samples of *A. pendulum* were distinguished using 11 ISSR primers, and their relationship was elucidated. The results indicated a relatively high level of genetic diversity in both *A. flavum* (*H*_e_ = 0.254, *I* = 0.395, *PPB* = 95.85%) and *A. pendulum* (*H*_e_ = 0.291, *I* = 0.445, *PPB* = 94.58%), compared with other *Aconitum* species (*A. firmum H*_e_ = 0.209, *PPB* = 50.30%; *A. lasiocarpum H*_e_ = 0.196, *PPB* = 43.00% [49]; *A. bucovinense H*_e_ = 0.080, *I* = 0.148, *PPB* = 69.77% [50]). We may state several possible reasons for the high genetic diversity of *A. flavum* and *A. pendulum*, which are the dominant species in high-altitude meadows. Firstly, the wide distribution range and long lifespans of *A. flavum* and *A. pendulum* contribute to their high genetic diversity. They are long-lived perennial herbaceous plants that are distributed in Qinghai, Gansu, Nei Mongol, Southern Ningxia, northwestern Sichuan, and northern Tibet [1]. Secondly, the genetic diversity of plants is also affected by their mating systems. Previous studies have indicated that the breeding system of *A. flavum* involves outcrossing determined by pollen–ovule ratios [51]. In addition, a S–RNase-based self-incompatibility system was found in *A. pendulum* by the authors of Li et al. [52]. We may say, then, that an outcrossing breeding system contributes to the high genetic diversity of the two species. It should also be stated that these species can produce many winged seeds which are spread by wind. Moreover, they can reproduce not only from seeds [53] but also by means of vegetative reproduction [54]. Finally, we note that, in recent years, due to both artificial introduction and natural reproduction, the distribution range of *A. flavum* and *A. pendulum* has expanded. This has contributed to a greater flow of genes between populations, resulting in a still higher level of genetic diversity.

## 4. Materials and Methods

### 4.1. Plant Materials

A total of 268 individuals from six populations were collected from Qinghai Province in China during July and August 2020 (Table 2). Specifically, 99 individuals of population DWM and 100 individuals of population QHL were used as samples to establish a species identification method. The remaining 69 individuals, from DWB, BM, GQ, and SL populations, were used as blind samples to validate the reliability of the ISSR marker. Healthy young leaves collected from individuals were dried in silica gel before genomic DNA extraction. All voucher specimens were deposited at room temperature in the Herbarium of the Northwest Institute of Plateau Biology (HNWP), Chinese Academy of Sciences. Then, they were used for microscopic morphology observation in our laboratory.

### 4.2. Microscopic Morphological Observation

The hairs on the pedicels of *A. flavum* and *A. pendulum* specimens were observed with a stereoscopic microscope (SteREO Discovery V12, Zeiss, Oberkochen, Germany) and photographed in our laboratory, during June and July 2021. In total, 140 voucher specimens (only the hairs on the pedicels) were observed under the microscope.

### 4.3. Genomic DNA Extraction

In our laboratory, genomic DNA was extracted using the modified CTAB method [55,56] in 2021 and 2022. The concentration and purity of the extracted DNA were determined by spectrophotometer using NanoDrop 2000c (Thermo Scientific, Waltham, MA, USA) equipment and 0.8% agarose gel. The isolated genomic DNA was diluted to 30 ng/µL and stored at −20 °C for ISSR amplification.

### 4.4. ISSR Amplification

One hundred primers from the University of British Columbia primer set 9 (University of British Columbia, primer set #9) were initially screened for PCR amplification, and eleven primers that generated clear and reproducible banding patterns were chosen for the final analysis. PCR amplifications were conducted in a 20 µL reaction volume containing 30 ng of genomic DNA, 2 µL of 10 × PCR buffer (Mg^2+^ plus), 0.25 mM of dNTP, 10 µM of primer, and 0.6 U of Taq DNA polymerase (TaKaRa Biotech Co., Ltd., San Jose, CA, USA). ISSR–PCR amplifications were conducted using a C1000 Touch Thermal Cycler (Bio-Rad, Hercules, CA, USA). The amplification conditions were as follows: an initial denaturation step at 95 °C for 4 min, followed by 38 cycles of denaturation at 95 °C for 30 s, annealing at 50–60 °C for 45 s (refer to Table S1 for specific details), and extension at 72 °C for 90 s. A final extension step at 72 °C for 7 min was then performed. The amplified products were separated on a 1.2% agarose gel and visualized using the ChemiDoc^TM^ MP Imaging System (Bio-Rad, Hercules, CA, USA). To ensure the reliability of the ISSR, two replicates for each PCR were produced in the lab.

### 4.5. Data Analysis

Only clearly identifiable and reproducibly amplified ISSR bands were designated as present (1) or absent (0). The resulting binary data matrix was subjected to analysis using POPGENE 1.32 version [57] to estimate the level of genetic diversity assuming the Hardy–Weinberg equilibrium. Genetic diversity within and among species was measured by the percentage of polymorphic bands (*PPB*), the observed number of alleles (*N*_a_), the effective number of alleles (*N*_e_), Nei’s gene diversity (*H*_e_) [58], and Shannon’s information index (*I*). Gene differentiation between species was assessed by the coefficient of gene differentiation (*G*_st_), and gene flow (*N*_m_) was assessed by *G*_st_ [59]. In order to test the genetic relationship between individuals, an unweighted pair-group method with arithmetic average (UPGMA) dendrogram was constructed based on the Jaccard coefficient using the program NTSYSpc version 2.2 [60]. PCoA (principal coordinates analysis) based on the Jaccard coefficient was performed to ordinate relationships among individuals of *A. flavum* and *A. pendulum*.

To correct the possible bias in the Hardy–Weinberg equilibrium, the Bayesian genetic diversity (*H*_B_) and population differentiation (*θ*_B_) were also calculated using HICKORY version 1.1 with the Bayesian method [61]. Using this Bayesian approach, neither the Hardy–Weinberg equilibrium within populations nor the treatment of multilocus ISSR phenotypes as haplotypes were assumed [62,63], but full advantage could be taken of the information provided by dominant markers. Several runs were carried out with default sample parameters (burnin = 5000, sample = 100,000, thin = 20) to ensure consistency of results. Model selection was based on the Deviance Information Criterion (DIC). Models with smaller DICs are preferred. In addition, we used two Bayesian methods to determine the genetic structures of *A. flavum* and *A. pendulum*. In the first method, population structures within *A. flavum* and *A. pendulum* samples were inferred using a Bayesian model clustering algorithm implemented in the computer program STRUCTURE version 2.3 [64]. This method uses a Markov Chain Monte Carlo (MCMC) algorithm to cluster individuals into populations based on multi-locus genotype data [65]. We tested the assignment of individuals into one to six genetic clusters (*K* = 1–6) using the admixture model with correlated allele frequencies. The analysis of each cluster consisted of 8 independent runs of 10,000 MCMC replicates following an initial burn-in of 10,000. To estimate the number of clusters, we used the *ΔK* [65] and the Ln Pr (X|K) plot methods [66], both calculated with the STRUCTURE HARVESTER [67] online software (http://taylor0.biology.ucla.edu/structureHarvester/). The algorithm used by STRUCTURE may be poorly suited for inferring the number of genetic clusters in a data set that has an isolation by distance relationship [65,68]. Therefore, we used BAPS (Bayesian analysis of population structure) version 6.0 [69,70], based on the Bayesian clustering method, to confirm the STRUCTURE result. In contrast with STRUCTURE, this method uses stochastic optimization to infer the genetic structure [68]. The BAPS program estimates the structure of populations by clustering individuals into groups. We considered individuals from one locality as one population sample (one group). Ten independent repetitions for each *K* from 1 to 2 were carried out. Arlequin version 3.1 was used to calculate the genetic differentiation index (*F*_st_) to further reveal the pattern of genetic differentiation between populations [71]. *F*_st_ is also a representation of population differentiation and genetic distance; the larger the index, the greater the differentiation. The statistical testing of variance components was conducted using nonparametric randomization tests with 1000 permutations.

## 5. Conclusions

In this study, we used a combination of morphological and molecular marker methods to confirm that *A. flavum* and *A. pendulum* are indeed two distinct species. Microscopic observations indicated that the spreading hairs on the inflorescence of *A. flavum* are appressed, while they are spread in *A. pendulum*. Additionally, *A. flavum* and *A. pendulum* were effectively identified using ISSR markers. Two primer combinations UBC808 and UBC853 successfully distinguished *A. flavum* from *A. pendulum*. Finally, a high level of genetic diversity in *A. flavum* and *A. pendulum* was detected via ISSR markers. This is the first time that ISSR molecular markers have been used to analyze the genetic diversity and structure of *A. flavum* and *A. pendulum*. The established identification system could provide a solid foundation for distinguishing *A. flavum* and *A. pendulum*.

## Figures and Tables

**Table 1:** Genetic diversity and differentiation in *A. flavum* and *A. pendulum* based on ISSR marker system.

Pop ID	*H*_e_ *	*I*	*PPB*	*G*_st_	*N*_m_	*F*_st_
DWM	0.230 ± 0.187	0.354 ± 0.257	80.31%	-	-	-
DWB	0.219 ± 0.190	0.334 ± 0.268	68.91%	-	-	-
BM	0.148 ± 0.195	0.221 ± 0.281	40.93%	-	-	-
*A. flavum*	0.254 ± 0.170	0.395 ± 0.223	95.85%	0.252	1.488	0.303
QHL	0.278 ± 0.171	0.424 ± 0.231	88.18%	-	-	-
GQ	0.127 ± 0.190	0.188 ± 0.276	33.00%	-	-	-
SL	0.129 ± 0.192	0.191 ± 0.276	34.48%	-	-	-
*A. pendulum*	0.291 ± 0.160	0.445 ± 0.209	94.58%	0.342	0.961	0.375

* *H*_e_—Nei’s gene diversity; *I*—Shannon’s information index ($=-\sum P_{i}\log_{2}P_{i}$, where $P_{i}$ is initial frequency for the *i*th allele); *PPB*—percentage of polymorphic bands; *G*_st_—coefficient of gene differentiation ($=\left(H_{t}-H_{s}\right)/H_{t}$); *N*_m_—gene flow ($=\left(1-G_{\mathrm{st}}\right)/2G_{\mathrm{st}}$); *F*_st_—fixation index ($=\left({\sigma_{a}}^{2}+{\sigma_{b}}^{2}\right)/\sigma^{2}$, where $\sigma_{a}$ and $\sigma_{b}$ are the expected value of the variance components between groups and within groups, respectively, with σ being the total of genetic variation).

**Table 2:** Sampling information for *A. flavum* and *A. pendulum*.

Collection Site	Population	Longitude (E)	Latitude (N)	Altitude (m) ^2^	Sample Size	Material
Dawu, Maqin county, Golog Prefecture	DWM ^1^	100°17′3″	34°25′50″	3762	99	Identification
Dawu, Maqin county, Golog Prefecture	DWB	100°17′3″	34°25′50″	3762	20	Verification
Banma county, Golog Prefecture	BM	100°43′22.80″	32°43′22.80″	4114	16	Verification
Gangcha county, Haibei Prefecture	QHL	100°14′48″	37°25′22″	3510	100	Identification
Guoqing pasture, Yushu city, Yushu Prefecture	GQ	96°51′17.52″	32°58′48.60″	4393	18	Verification
Sulu, Zaduo county, Yushu Prefecture	SL	95°9′55.14″	32°33′19.20″	4518	15	Verification

^1^ DWM—Meadow of Dawu; DWB—Bareground of Dawu; BM—Banma; QHL—Gangcha; GQ—Guoqing pasture; SL—Sulu; ^2^ altitude (m)—meters above sea level.

## Data Availability

Data are available upon request from the corresponding authors.

## Supplementary Materials

The following supporting information can be downloaded at: https://www.mdpi.com/article/10.3390/plants13060885/s1, Figure S1: Results of BAPS analysis for two species of *Aconitum*; Figure S2: UPGMA clustering on verification of blind individuals (UBC808 and UBC853): (A) blind individuals from DWB and BM; (B) blind individuals from GQ and SL. Green represents *A. flavum* individuals; red represents *A. pendulum* individuals; Figure S3: ISSR profiles of *A. flavum* and *A. pendulum* generated with primer UBC853: (A) DWB population; (B) QHL population; M = 100 bp + 200 bp TaKaRa DNA Ladder; Table S1: Summary of banding profile and polymorphism revealed by 11 ISSR primers; Table S2: Analysis of molecular variance (AMOVA) for ISSR variation surveyed in six populations of *A. flavum* (3) and *A. pendulum* (3); Table S3: Genetic differentiation among species calculated using different Bayesian approaches.

## References

[1] Li L.Q., Kadota Y. (2001). Aconitum Linnaeus. Flora of China.

[2] Xiao P.G., Wang F.P., Gao F., Yan L.P., Chen D.L., Liu Y. (2006). A pharmacophylogenetic study of *Aconitum* L. (Ranunculaceae) from China. Acta. Phytotaxon. Sin..

[3] Ma L., Gu R., Tang L., Chen Z.E., Di R., Long C. (2015). Important poisonous plants in Tibetan ethnomedicine. Toxins.

[4] Han L.H., Shang Z.H., Ren G.H., Wang Y.L., Ma Y.S., Li X.L., Long R.J. (2011). The response of plants and soil on black soil patch of the Qinghai–Tibetan Plateau to variation of bare-patch areas. Acta Pratac. Sin..

[5] Shang Z.H., Tang Y., Long R.J. (2011). Allelopathic effect of *Aconitum pendulum* (Ranunculaceae) on seed germination and seedlings of five native grass species in the Tibetan Plateau. Nord J. Bot..

[6] Li C.Y., Zhou Z., Xu T., Wang N.Y., Tang C., Tan X.Y., Feng Z.G., Zhang Y., Liu Y. (2022). *Aconitum pendulum* and *Aconitum flavum*: A narrative review on traditional uses, phytochemistry, bioactivities and processing methods. J. Ethnopharmacol..

[7] Tiwari S., Acharya P., Solanki B., Sharma A.K., Rawat S. (2023). A review on efforts for improvement in medicinally important chemical constituents in *Aconitum* through biotechnological interventions. 3 Biotech.

[8] Salick J., Byg A., Amend A., Gunn B., Law W., Schmidt H. (2006). Tibetan medicine plurality. Econ. Bot..

[9] El–Shazly M., Tai C.J., Wu T.Y., Csupor D., Hohmann J., Chang F.R., Wu Y.C. (2016). Use, history, and liquid chromatography/mass spectrometry chemical analysis of *Aconitum*. J. Food Drug. Anal..

[10] Chinese Pharmacopoeia Commission (1995). Pharmacopoeia Standards of the Ministry of Health of the People’s Republic of China: Tibetan Medicine.

[11] Zhang Y., Zhong G. (2005). Qiang Medicine.

[12] Duo J.C., Li C.X., Xu X.L., Feng H.S., Song W.Z., Ma S.Z. (2022). Research progress and prospects of pustainable utilization, chemical composition and pharmacological effects of the resource of *Aconitum Flavum* Hand.-Mazz. China Wild Plant Resour..

[13] Yang Q.E. (1999). Taxonomic notes on some species of *Aconitum* L. (Ranunculaceae) from Yunnan, China. J. Syst. Evol..

[14] Luo Y., Yang Q.E. (2005). Taxonomic revision of *Aconitum* (Ranunculaceae) from Sichuan, China. J. Syst. Evol..

[15] Wang W.C. (1979). Angiospermae. Flora of China.

[16] Nezami E., Gallego P.P. (2023). History, phylogeny, biodiversity, and new computer-based tools for efficient micropropagation and conservation of Pistachio (*Pistacia* spp.) germplasm. Plants.

[17] Huang S.Q., Zhang Y.Y., Li Y.Z., Fan H., Huang W.L., Deng C., Wang W., Song X.M. (2020). Research progress of *Aconitum szechenyianum* Gay. China Wild Plant Resour..

[18] Stavridou E., Lagiotis G., Karapetsi L., Osathanunkul M., Madesis P. (2020). DNA fingerprinting and species identification uncovers the genetic diversity of Katsouni Pea in the Greek islands Amorgos and Schinoussa. Plants.

[19] Munankarmi N.N., Rana N., Bhattarai T., Shrestha R.L., Joshi B.K., Baral B., Shrestha S. (2018). Characterization of the genetic diversity of Acid Lime (*Citrus aurantifolia* (Christm.) Swingle) cultivars of Eastern Nepal using Inter-Simple Sequence Repeat markers. Plants.

[20] Vieira J.P.S., Selbach-Schnadelbach A., Braz M., Ribeiro P.L., van den Berg C., Oliveira R.P. (2023). Coalescent-Based Species delimitation in herbaceous Bamboos (Bambusoideae, Olyreae) from Eastern Brazil: Implications for Taxonomy and conservation in a group with weak morphological divergence coupled with low genetic diversity. Plants.

[21] Reddy M.P., Sarla N., Siddiq E.A. (2002). Inter simple sequence repeat (ISSR) polymorphism and its application in plant breeding. Euphytica.

[22] Jedrzejczyk I., Rewers M. (2018). Genome size and ISSR markers for *Mentha* L. (Lamiaceae) genetic diversity assessment and species identification. Ind. Crop. Prod..

[23] Ma S., Khayatnezhad M., Minaeifar A.A. (2021). Genetic diversity and relationships among *Hypericum* L. species by ISSR markers: A high value medicinal plant from Northern of Iran. Caryologia.

[24] Li A., Ma M., Li H., He S., Wang S. (2023). Genetic diversity and population differentiation of a Chinese endangered plant *Ammopiptanthus nanus* (M. Pop.) Cheng f. Genes.

[25] Yang Y., Tang J., Zou R., Luo Y., Deng Z., Li D., Chai S., Wei X. (2023). The genetic diversity and genetic structure of the germplasm resources of the medicinal orchid plant *Habenaria dentata*. Genes.

[26] Zietkiewicz E., Rafalski A., Labuda D. (1994). Genome fingerprinting by Simple Sequence Repeat (SSR)-anchored polymerase chain reaction amplification. Genomics.

[27] Contreras R., van den Brink L., Burgos B., Gonzalez M., Gacitua S. (2020). Genetic characterization of an endangered Chilean endemic species, *Prosopis burkartii* Munoz, reveals its hybrids parentage. Plants.

[28] Araujo F.S., Bruno R.A., Arriel N.H.C., de Medeiros E.P., de Lima L.M., de Souza M.A., de Andrade A.P., Silva R.A.R., Felix F.C., Belarmino K.S. (2023). Genetic polymorphism detection in brazilian perennial cottons (*Gossypium* spp.) using an ISSR marker system and its application for molecular interspecific differentiation. Mol. Biol. Rep..

[29] Cheng J., Hu D., Liu Y., Zhang Z., Khayat–Nezhad M. (2021). Molecular identification and genetic relationships among *Alcea* (Malvaceae) species by ISSR Markers: A high value medicinal plant. Caryologia.

[30] Akhtar N., Hafiz I.A., Hayat M.Q., Potter D., Abbasi N.A., Habib U., Hussain A., Hafeez H., Bashir M.A., Malik S.I. (2021). ISSR-based genetic diversity assessment of genus *Jasminum* L. (Oleaceae) from Pakistan. Plants.

[31] Meng F., Wang R., Peng M., Wang C., Wang Z., Guan F., Li Y. (2015). Evaluation of genetic diversity among Kongpo Monkshood (*Aconitum kongboense* L.) germplasm accessions revealed by Inter Simple Sequence Repeat markers. Hortscience.

[32] Gao F.C., Sun Y., Zhang J., Zhang F. (2014). ISSR analysis for genetic polymorphism of *Aconitum leucostomum* from different habitats. J. Chin. Med. Mater..

[33] Luo Q., Ma D.W., Wang Y.H. (2006). ISSR identification of genetic diversity in *Aconitum carmichaeli*. Chin. Tradit. Herbal Drugs.

[34] Luo Y., Zhang F.M., Yang Q.E. (2005). Phylogeny of *Aconitum* subgenus *Aconitum* (Ranunculaceae) inferred from ITS sequences. Plant Syst. Evol..

[35] Cui Y., Qi P.C., Wang X.L. (2018). Cladistic systematics of *Aconitum* in Gansu Province. Rural Econ. Sci.–Technol..

[36] Li Q. (2022). Species divergence of *Aconitum pendulum* and *Aconitum flavum*. Master’s Thesis.

[37] Ren L.M. (2019). A taxonomical study of Trib. *Delphineae* Warming (Ranunculaceae) from Gansu. Master’s Thesis.

[38] Kita Y., Ueda K., Kadota Y. (1995). Molecular phylogeny and evolution of the Asian *Aconitum* subgenus *Aconitum* (Ranunculaceae). J. Plant. Res..

[39] Liu Y., Yu S.H., You F.M. (2020). Characterization of the complete chloroplast genome of *Aconitum flavum* (Ranunculaceae). Mitochondrial DNA B.

[40] Wang Z.H., Li Y.Q. (2020). Characterization of the complete chloroplast genome of *Aconitum pendulum* (Ranunculaceae), an endemic medicinal herb. Mitochondrial DNA B.

[41] Jabbour F., Renner S.S. (2012). A phylogeny of Delphinieae (Ranunculaceae) shows that *Aconitum* is nested within *Delphinium* and that Late Miocene transitions to long life cycles in the Himalayas and Southwest China coincide with bursts in diversification. Mol. Phylogenet. Evol.

[42] Tamura M. (1995). Die Natürlichen Pflanzenfamilien, Zweite Auflage.

[43] Kong H.H., Gao Q., Luo Y., Yang Q.E. (2013). Seed morphology in some Chinese species of *Aconitum* (Ranunculaceae) and its systematic implications. Plant Divers. Resour..

[44] Li Q., Xia M., Yu J., Chen S., Zhang F. (2022). Plastid genome insight to the taxonomic problem for *Aconitum pendulum* and *A. flavum* (Ranunculaceae). Biologia.

[45] Xia C., Wang M., Guan Y., Li J. (2022). Comparative analysis of the chloroplast genome for *Aconitum* species: Genome structure and phylogenetic relationships. Front. Genet..

[46] Boydak M., Teker T., Gazdagli A., Thanos C.A., Caliskan S., Kaltsis A., Tozlu E.C., Fournaraki C., Albayrak G. (2021). ISSR genotyping of *Phoenix theophrasti* natural populations in Turkey and Crete (Greece) and *P. dactylifera*. Nord. J. Bot..

[47] Liu S., Wang Y., Song Y., Khayatnezhad M., Minaeifar A.A. (2021). Genetic variations and interspecific relationships in *Salvia* (Lamiaceae) using SCoT molecular markers. Caryologia.

[48] Tomasello S., Alvarez I., Vargas P., Oberprieler C. (2015). Is the extremely rare Iberian endemic plant species *Castrilanthemum debeauxii* (Compositae, Anthemideae) a ‘living fossil’? Evidence from a multi-locus species tree reconstruction. Mol. Phylogenet. Evol..

[49] Sutkowska A., Boron P., Mitka J. (2013). Natural hybrid zone of *Aconitum* species in the Western Carpathians: Linnaean taxonomy and ISSR fingerprinting. Acta Biol. Cracov. Bot..

[50] Boron P., Zalewska-Galosz J., Sutkowska A., Zemanek B., Mitka J. (2011). ISSR analysis points to relict character of *Aconitum bucovinense* Zapal. (Ranunculaceae) at the range margin. Acta Soc. Bot. Pol..

[51] Zeng H., Tong Z., Qu X., Jin X., Chen H., Wang J. (2009). Studies on the ecological and biological characteristics of reproductive module of *Aconitum flavum* Hand.-Mazz. growth in Liu Pan Shan area. Chin. Hortic. Abstr..

[52] Li X., Geng T., Wang Y., Qian T., Zhang Y., Zhao F., Sun K., Zhang H. (2021). Mining and analysis of the self-incompatibility S gene in *Aconitum pendulum* N. Busch based on RNA-seq. Plant Sci. J..

[53] Zhang Y.X., Tang J.N., Xu Q. (2011). Study on characteristics of flower organs and flowering and fruiting habits of *A. flavum* Hand.-Mazz. in Liupanshan. J. Anhui Agri. Sci..

[54] Ni D.W., Chen H.G. (2022). Influence of cultivation techniques and cultivation years on the quality of *A. Pendulum*. Agri. Sci. Technol. Inf..

[55] Zhang H.X., Li W.J., Wang J., He R., Li Y., Hu Y.P. (2022). Optimization of DNA isolation and ISSR–PCR system of *Aconitum flavum* Hand.-Mazz. Mol. Plant Breed..

[56] Doyle J.J., Doyle J.L. (1987). A rapid DNA isolation procedure for small quantities of fresh leaf tissue. Phytochem. Bull..

[57] Yeh F.C., Yang R.C., Boyle T. (1999). POPGENE, Microsoft Window-Based Freeware for Population Genetic Analysis, version 1.32.

[58] Nei M. (1973). Analysis of gene diversity in subdivided populations. Proc. Natl. Acad. Sci. USA.

[59] McDermott J.M., McDonald B.A. (1993). Gene flow in plant pathosystems. Annu. Rev. Phytopathol..

[60] Rohlf F.J. (2009). NTSYS–pc: Numerical Taxonomy and Multivariate Analysis System, version 2.2.

[61] Holsinger K.E., Lewis P.O., Dey D.K. (2002). A Bayesian approach to inferring population structure from dominant markers. Mol. Ecol..

[62] Liu J.M., Wang L., Geng Y., Wang Q., Luo L., Zhong Y. (2006). Genetic diversity and population structure of *Lamiophlomis rotata* (Lamiaceae), an endemic species of Qinghai–Tibet Plateau. Genetica.

[63] Hu Y., Wang L., Xie X., Zhang H., Yang J., Li Y. (2014). Genetic variation in cultivated rhubarb (*Rheum tanguticum* Maxim. ex Balf.) and the relationship with their wild relatives in China revealed by ISSR markers. Plant Syst. Evol..

[64] Pritchard J.K., Stephens M., Donnelly P. (2000). Inference of population structure using multilocus genotype data. Genetics.

[65] Evanno G., Regnaut S., Goudet J. (2005). Detecting the number of clusters of individuals using the software STRUCTURE: A simulation study. Mol. Ecol..

[66] Pritchard J.K., Wen W., Falush D. (2010). Documentation for STRUCTURE Software.

[67] Earl D.A., von Holdt B.M. (2012). STRUCTURE HARVESTER: A website and program for visualizing STRUCTURE output and implementing the Evanno method. Conserv. Genet. Resour..

[68] Lavandero B., Miranda M., Ramirez C.C., Fuentes-Contreras E. (2009). Landscape composition modulates population genetic structure of *Eriosoma lanigerum* (Hausmann) on *Malus domestica* Borkh in central Chile. Bull. Entomol. Res..

[69] Corander J., Waldmann P., Sillanpää M.J. (2003). Bayesian analysis of genetic differentiation between populations. Genetics.

[70] Corander J., Marttinen P., Siren J., Tang J. (2008). Enhanced Bayesian modelling in BAPS software for learning genetic structures of populations. BMC Bioinform..

[71] Raymond M., Rousset F. (1995). An exact test for population differentiation. Evolution.

