# Supplementary material for: Species Identification and Genetic Diversity Analysis of Medicinal Plants Aconitum pendulum Busch and Aconitum flavum Hand.-Mazz
Source: Plants (Basel). 2024 Mar 19;13(6):885. doi: 10.3390/plants13060885 (PMC10976075; doi:10.3390/plants13060885)
Supplement: Supplementary file 1 [file plants-13-00885-s001.zip › plants-2833188-supplementary.pdf]

Supplementary Material

Jing Sun [1,2], Qing Sun [1,2], Xin Li [1,2], Wenjing Li [3], Yi Li [1], Yubi Zhou [1] and Yanping Hu [1,*]

[1] Qinghai Provincial Key Laboratory of Qinghai–Tibet Plateau Biological Resources, Northwest Institute of Plateau Biology, Chinese Academy of Sciences, Xining 810008, China; sunjing21@mails.ucas.ac.cn (J.S.); sunqing22@mails.ucas.ac.cn (Q.S.); lixin2311@mails.ucas.ac.cn (X.L.); liyi@nwipb.cas.cn (Y.L.); ybzhou@nwipb.cas.cn (Y.Z.)

[2] University of Chinese Academy of Sciences, Beijing 100049, China

[3] Scientific Research and Popularization Base of Qinghai–Tibet Plateau Biology, Qinghai Provincial Key Laboratory of Animal Ecological Genomics, Xining 810008, China; wjli@nwipb.cas.cn

* Correspondence: yphu@nwipb.cas.cn

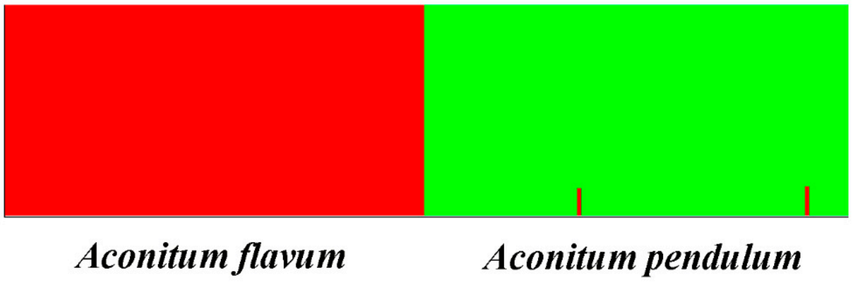

Figure S1. Results of BAPS analysis for two species of *Aconitum*.

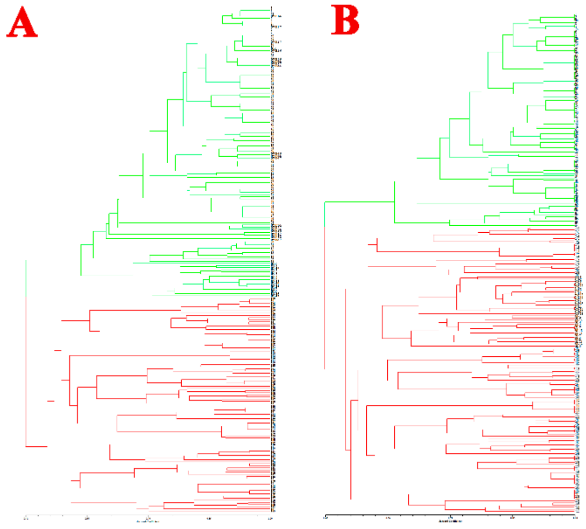

Figure S2. UPGMA clustering on verification of blind individuals (UBC808 and UBC853): (**A**) blind individuals from DWB and BM; (**B**) blind individuals from GQ and SL. Green represents *A. flavum* individuals; red represents *A. pendulum* individuals.

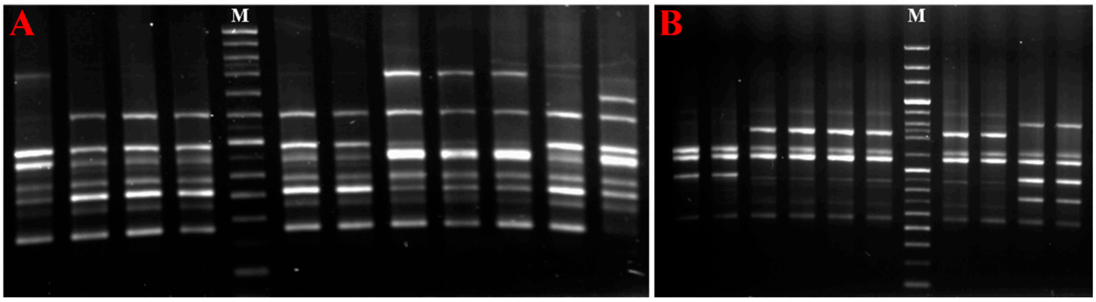

Figure S3. ISSR profiles of *A. flavum* and *A. pendulum* generated with primer UBC853: (**A**) DWB population; (**B**) QHL population; M = 100 bp + 200 bp TaKaRa DNA Ladder.

Table S1. Summary of banding profile and polymorphism revealed by 11 ISSR primers.

Primer ID	Primer sequence (5'-3')	TM (℃)	*A. flavum*				*A. pendulum*			
			TNB	NPB	*PPB* (%)	Size (bp)	TNB	NPB	*PPB* (%)	Size (bp)
UBC807	AGAGAGAGAGAGAGAGT	51.8	14	13	92.86	350-1,200	17	14	82.35	300-1,200
UBC808	AGAGAGAGAGAGAGAGC	59.6	17	16	94.12	300-1,700	19	17	89.47	300-1,700
UBC811	GAGAGAGAGAGAGAGAC	57.4	14	11	78.57	300-1,300	18	15	83.33	300-1,300
UBC812	GAGAGAGAGAGAGAGAA	52	16	14	87.50	400-1,800	17	15	88.24	400-1,800
UBC824	TCTCTCTCTCTCTCTCG	52	18	18	100.00	400-2,000	20	20	100.00	400-2,000
UBC825	ACACACACACACACACT	53	22	22	100.00	400-2,000	20	20	100.00	400-2,000
UBC826	ACACACACACACACACC	60	19	19	100.00	300-2,000	16	16	100.00	300-2,000
UBC846	CACACACACACACACART	52	19	19	100.00	300-1,800	20	20	100.00	450-1,800
UBC853	TCTCTCTCTCTCTCTCRT	50	13	12	92.31	550-1,900	16	16	100.00	550-1,900
UBC887	DVDTCTCTCTCTCTCTC	52	20	20	100.00	350-2,000	21	21	100.00	350-2,000
UBC890	VHVGTGTGTGTGTGTGT	56	21	21	100.00	350-2,000	19	18	94.74	350-2,000
Total	-	-	193	185	95.85	300-2,000	203	192	94.58	300-2,000
Means	-	-	17.55	16.82	95.85	-	18.45	17.45	94.58	-

D= (A, G, T); H= (A, C, T); R= (A, G); V= (A, C, G); Total Number of amplified Bands (TNB); Number of Polymorphic Bands (NPB), Percentage of Polymorphic Bands (*PPB*).

Table S2. Analysis of molecular variance (AMOVA) for ISSR variation surveyed in six populations of *A. flavum* (3) and *A. pendulum* (3)

Source of variation	*d.f.*	SSD	MSD	Variation components	Total variation (%)	*P* value[a]
A. flavum						
Between populations	2	596.373	298.187	9.59849	30.34	$P < 0.001$
Within populations	132	2,908.545	22.034	22.03443	69.66	$P < 0.001$
A. pendulum						
Between populations	2	865.13	432.565	15.17223	37.48	$P < 0.001$
Within populations	130	3,290.359	25.310	25.31045	62.52	$P < 0.001$

d.f.: Degrees of freedom, SSD: sum of squares, MSD: mean squared deviation;

[a]: Significance tests after 1,000 permutations.

Table S3. Genetic differentiation among species calculated using different Bayesian approaches.

Model	populations differentiation (θ_B)					inbreeding coefficient (*f*)					DIC
	Mean	SD	2.50%	50%	97.50%	Mean	SD	2.50%	50%	97.50%	
Full model	0.265	0.032	0.201	0.266	0.324	0.554	0.272	0.050	0.567	0.976	2,517.43
$f = 0$ model	0.207	0.019	0.173	0.206	0.248	-	-	-	-	-	2,549.28
$\theta_B = 0$ model	-	-	-	-	-	0.981	0.018	0.932	0.987	0.999	10,255.70
f free model	0.288	0.027	0.237	0.288	0.341	0.493	0.291	0.023	0.488	0.977	3,651.48

Hickory 1.1 Software uses the inbred coefficient (*f*), f = Full Model, 0 (assuming no inbreeding), free model and $\theta_B = 0$ model (assuming no population structure). The model calculates genetic differentiation and the genetic structure parameters of *A. flavum* and *A. pendulum*. Model selection was based on the Deviation Information Criterion (DIC). Models with smaller DICs are preferred.
